# Conventional inactivated bivalent H5/H7 vaccine prevents viral localization in muscles of turkeys infected experimentally with low pathogenic avian influenza and highly pathogenic avian influenza H7N1 isolates

**DOI:** 10.1080/03079450802061124

**Published:** 2008-07-23

**Authors:** Anna Toffan, Maria Serena Beato, Roberta De Nardi, Elena Bertoli, Annalisa Salviato, Giovanni Cattoli, Calogero Terregino, Ilaria Capua

**Affiliations:** OIE, FAO and National Reference Laboratory for Avian Influenza and Newcastle Disease, OIE Collaborating Centre for Epidemiology, Training and Control of Emerging Avian Diseases, Istituto Zooprofilattico Sperimentale delle Venezie, Viale dell’Università 10, 35020 Legnaro, Padova, Italy

## Abstract

Highly pathogenic avian influenza (HPAI) viruses cause viraemia and systemic infections with virus replication in internal organs and muscles; in contrast, low pathogenicity avian influenza (LPAI) viruses produce mild infections with low mortality rates and local virus replication. There is little available information on the ability of LPAI viruses to cause viraemia or on the presence of avian influenza viruses in general in the muscles of infected turkeys. The aim of the present study was to determine the ability of LPAI and HPAI H7N1 viruses to reach muscle tissues following experimental infection and to determine the efficacy of vaccination in preventing viraemia and meat localization. The potential of infective muscle tissue to act as a source of infection for susceptible turkeys by mimicking the practice of swill-feeding was also investigated. The HPAI virus was isolated from blood and muscle tissues of all unvaccinated turkeys; LPAI could be isolated only from blood of one bird and could be detected only by reverse transcriptasepolymerase chain reaction in muscles. In contrast, no viable virus or viral RNA could be detected in muscles of vaccinated/challenged turkeys, indicating that viral localization in muscle tissue is prevented in vaccinated birds.

## Introduction

Avian influenza (AI) represents one of the major concerns for public health that has emerged in recent times. Over the past decade a sharp increase in the number of outbreaks in birds, especially poultry, has occurred, with a high economic impact on the commercial poultry sector. Moreover, fears about the potential zoonotic risk have concurred to reduce market demand for poultry products, both in affected countries and in non-affected countries, increasing the crisis for the poultry industry and affecting employment rates in some countries (Doyle & Erickson, 2006).

The occurrence of major AI outbreaks between 1999 and 2004, caused by viruses of the H7 subtype, have stimulated the revision of the definition of AI for statutory and trade purposes (Capua & Alexander, 2006). One of the main changes in current European Union legislation and in World Organization for Animal Health (OIE) guidelines is that the use of vaccination is seen as a potentially useful aid in the control of AI infections.

The issue of trading meat, obtained from vaccinated animals, has been the subject of international debate for a significant amount of time, and the consensus reached is that meat and products obtained from vaccinated animals can be traded, provided the animals can be shown not to be infected at the time of slaughter. Although several systems exist to differentiate infected from vaccinated animals (Capua & Cattoli, 2007), a risk that a vaccinated/infected flock may escape surveillance systems in place cannot be ruled out, particularly during the early phases of infection. This raises concerns for the role that potentially infected meat may play in the spread of infection to avian and non-avian hosts.

In addition to the H7N1 and H7N3 epidemic waves of 1999 to 2003, ongoing surveillance programmes in Italy have shown that low pathogenicity avian influenza (LPAI) viruses of the H7 subtype appear to be prevalent in the wild bird population and have also been detected in backyard flocks (Terregino *et al.*, 2007). Field and laboratory evidence indicates that turkeys are particularly susceptible to AI virus infections (Werner *et al.*, 2000; Capua *et al.*, 2003; Tumpey *et al.*, 2004; McNally *et al.*, 2006), requiring minimal doses of field virus to develop infection.

Highly pathogenic avian influenza (HPAI) virus infections in *Galliformes* species produce a systemici disease causing viraemia with viral replication and localization in organs and muscles (Mo *et al.*, 1997; Perkins & Swayne, 2001; Starick & Werner, 2003; Swayne & Beck, 2005). In contrast, LPAI virus infections usually cause milder conditions, which can be complicated by other pathogens (Capua & Marangon, 2000; Kishida *et al.*, 2004). For LPAI viruses, viral replication is allegedly restricted to the respiratory and gastrointestinal tracts (Alexander, 2000), but little is known on the ability of these viruses to replicate in other tissues and organs including muscles. For this reason we carried out an investigation to detect the presence of viral RNA and live virus in meat collected from vaccinated and unvaccinated turkeys experimentally infected with HPAI and LPAI viruses of the H7 subtype. Additional information on the risk posed by infected meat on spread of infection through swill feeding was also generated.

## Materials and Methods

### Vaccine and vaccination scheme

Chickens and turkeys were immunized with a commercially available inactivated bivalent vaccine containing the following strains: H5N9 LPAI (A/chicken/Italy/22A/1998) and H7N1 LPAI (A/chicken/Italy/1067/1999). The vaccination scheme consisted of two doses of 0.5 ml administered by the subcutaneous route in the back of the neck at 19 days of age and 3 weeks later (at 40 days of age). Both chickens and turkeys were given the same vaccination protocol.

Serum samples collected from vaccinated birds before each vaccination and before challenge were tested by haemagglutination inhibition (HI) tests for antibodies to the H7 subtype. The log_2_ geometric mean titre of the serum of vaccinated turkeys was calculated.

### Viruses

Experimental infection of turkeys was carried out with two H7N1 viruses: A/turkey/Italy/3675/1999 (LPAI) and A/turkey/Italy/4580/1999 (HPAI).

Viruses were titrated in 9-day-old to 10-day-old embryonated eggs from specific pathogen free (SPF) domestic fowls, using 10-fold dilutions and five eggs per dilution. The median egg infectious dose (EID_50_) was calculated according to the Reed and Muench formula (Reed & Muench, 1938).

## Experimental Design

### Animals

Commercially available 1-day-old poults were used in this study. Experimental birds originating from a parent flock that was serologically negative by agar gel immunodiffusion (AGID) test and enzyme-linked immunosorbent assay (ELISA) tests and by real-time reverse transcriptase-polymerase chain reaction (RRT-PCR) on cloacal swabs were used. One-day-old SPF chicks were used in the swill-feeding experiment.

All animals were identified by means of wing tags and received feed and water ad *libitum*. Birds were housed in negative-pressure, high-efficiency particulate air-filtered isolation cabinets for the duration of the experimental trial.

### Assessment of viral presence in muscles

Two groups of 15 vaccinated poults 6 to 7 weeks old and two groups of 15 unvaccinated hatch mates were used in the experimental trial. At 50 days of age (10 days after the second vaccination) all experimental groups were given 100 μl challenge virus containing 10^6^ EID_50_ oro-nasally. For clarity, each of the two challenge viruses was given to one vaccinated group and one unvaccinated group.

On days 1, 2, 3, 4 and 5 post infection (p.i.), blood was collected from each bird from the wing vein, mixed with anticoagulant (Alsever’s solution 1:1) and the establishment of viraemia was evaluated by RRTPCR. If blood samples yielded positive results, up to three birds from each group presenting viraemia were killed humanely on the day of testing. When blood samples yielded negative results and no animals showed clinical signs, three turkeys were killed humanely on a random basis; otherwise, those presenting clinical signs were sacrificed. In the case of any death, organs were collected on the day of death. Lungs, superficial and deep pectoral muscles and thigh muscles were collected from each killed or naturally deceased turkey.

### Avian influenza transmission-feeding test

For this experimental trial, two groups of five 7-week-old SPF chickens and two groups of five turkeys of the same age were used. One group of chickens and one group of turkeys were vaccinated as described above and two groups were left unvaccinated.

At 50 days of age, experimental groups of birds were fed with a meat homogenate prepared as described below and were observed daily for clinical signs. Tracheal and cloacal swabs were collected on days 3, 5 and 7 after administration of the homogenate. Serum samples were also collected on 7, 14 and 21 days post administration of the homogenate.

The infective meat homogenate was prepared with meat samples positive by virus isolation (VI) collected from unvaccinated turkeys infected with the HPAI virus. Muscles that yielded the highest viral titres were homogenized together with sterile quartz sand. The meat homogenate was further titrated in embryonated SPF eggs to ascertain the infectious dose. Two grams of the infective meat homogenate with a viral titre of 10^3.6^ EID_50_/0.1g were administered to each animal by the oral route. The infective meat homogenate was introduced directly into the oesophagus with a syringe in order to reduce the risk of inhalation and to ascertain the ingestion of the whole sample.

### Virus isolation

The organs collected were weighed (1 g) and homogenized with sterile quartz sand and phosphate-buffered solution (PBS) containing antibiotics and 20% glycerol (v/v) to make a 1:10 (w/v) suspension. Blood samples, collected for the evaluation of viraemia, were diluted 1:10 in PBS containing antibiotics to avoid the embryo toxicity of the anticoagulant. Tracheal and cloacal swabs were immersed in 1 ml PBS containing antibiotics. VI and titration were performed according to the EU diagnostic manual (Commission Decision, 2006).

### Real-time reverse transcriptase-polymerase chain reaction

Organs were weighed (0.1 g) and homogenized with sterile quartz sand in 1 ml sterile PBS to make 1:10 (w/v) dilution.

#### Extraction of RNA

Two-hundred microlitres of PBS suspension from homogenated organs, blood and swabs were used to extract the RNA using a commercial kit (High Pure^TM^ RNA extraction kit; Roche). RNA was eluted in a final volume of 60 μl containing 20 u Rnase Inhibitor (Applied Biosystems) and was stored at −80°C.

#### Real-time reverse transcriptase-polymerase chain reaction

Thirty microlitres of RNA solution were retrotranscribed with random hexamers in a final volume of 60 μl following the instructions of the kit (High Capacity® cDNA Archive kit; Applied Biosystems). Published primers and probes (Spackman *et al.*, 2002) targeting the M gene of type A influenza virus were applied for PCR; namely, forward primer M+25 and reverse primer M-124 at the optimized concentration of 300 nM each, and the specific fluorescent label probe M+64 was used at the final concentration of 250 nM. cDNA was amplified in a final volume of 25 μl using TaqMan® Universal PCR Master Mix. The PCR reaction was performed in a ABI Prism 7700 SDS apparatus (Applied Biosystem) with the following protocol: 2 min at 50°C and 10 min at 95°C followed by 40 cycles at 95°C for 10 sec and 60°C for 1 min. Samples with a threshold cycle value ≤35 were considered positive for influenza type A viral RNA based on internal validation trials.

### Serology

Type and subtype specific antibodies were detected by HI tests and a ELISA, developed in-house, which utilizes a monoclonal antibody against the nucleoprotein of type A influenza viruses. For the HI test, the detection of antibodies to the H7 and H5 subtypes of AI was performed using 4 haemagglutinating units of the antigens H5N9 (A/chicken/Italy/22A/1998) and H7N1 (A/chicken/Italy/1067/1999).

Sera collected from vaccinated animals were considered positive with an increase in antibody titre in HI tests ≥4 log_2_ compared with pre-infection titres. Naïve animals were considered positive with a serological titre ≥4 log_2_.

## Results

### Assessment of viral presence in muscles

#### Unvaccinated turkeys infected with HPAI virus

Owing to the clinical condition and subsequent high mortality rate post challenge, it was not possible to follow the experimental design for this group, and to collect samples until day 5 p.i. All unvaccinated, HPAI-infected turkeys showed severe clinical signs starting from day 1 p.i., with 100% mortality by day 4 p.i. (Table 1). On day 1 p.i., two out of 14 blood samples (one turkey was found dead) yielded positive results by RRT-PCR. On day 2 p.i., seven out of 11 blood samples were positive; and on day 3 p.i., blood from all the surviving birds yielded positive results (Tables 1 and 2). VI confirmed these results. Virus was detected by RRT-PCR and VI from lung and breast muscle samples of all dead and killed turkeys, and from thigh muscles of all but two birds killed humanely on day 1 p.i. (Table 2). The amount of virus varied between 10^1^and 10^4.38^ EID_50_/0.1 g in muscle tissues and from 10^1^ to 10^5.8^ EID_50_/0.1 ml in blood (Table 1).

**Table 1:** Clinical signs, schedule of death/sacrificing and time of appearance of viraemia by RRT-PCR of unvaccinated turkeys infected with HPAI virus A/turkey/Italy/4580/1999 (H7N1)

Turkey number	1 day p.i.	2 days p.i.	3 days p.i.	4 days p.i.
381	Healthy/killed humanely	–	–	–
382	Healthy/killed humanely viraemic (<10^1^)	–	–	–
383	Healthy	Sick viraemic (<10^1^)	Dead	–
384	Healthy	Sick	Sick viraemic (10^4.3^)	Dead
385	Healthy	Sick	Sick viraemic (10^3.8^)	Dead
386	Healthy	Dead viraemic (10^4.5^)	–	–
387	Healthy	Dead viraemic (10^5.8^)	–	–
388	Healthy/killed humanely viraemic (10^1.25^)	–	–	–
389	Healthy	Sick	Dead	–
390	Healthy	Sick viraemic (10^3.6^)	Sick viraemic (10^1.8^)	Dead
391	Healthy	Sick	Sick viraemic (10^4.6^)	Dead
392	Healthy	Sick viraemic (10^2.4^)	Dead	–
393	Healthy	Killed humanely viraemic (10^3.5^)	–	–
394	Healthy	Sick viraemic (10^1.7^)	Dead	–
395	Dead	–	–	–

Data in parentheses are the viral titre expressed as EID_50_/0.1 ml.

**Table 2:** RRT-PCR and VI results in unvaccinated turkeys infected with HPAI virus A/turkey/Italy/4580/1999 (H7N1)

Group	1 day p.i.	2 days p.i.	3 days p.i.	4 days p.i.	5 days p.i.
Virus isolation					
Breast	4/4	3/3	4/4	4/4	Not done
Thigh	2/4	3/3	4/4	4/4	Not done
Lung	4/4	3/3	4/4	4/4	Not done
Blood	2/14	7/11	4/4	Not done	Not done
RRT-PCR					
Breast	4/4	3/3	4/4	4/4	Not done
Thigh	2/4	3/3	4/4	4/4	Not done
Lung	4/4	3/3	4/4	4/4	Not done
Blood	2/14	7/11	4/4	Not done	Not done

Data presented as number positive/total.

#### Vaccinated turkeys infected with HPAI virus

No clinical signs were observed during the experimental trial. No virus was detected in blood and muscle samples by RRT-PCR or VI. Virus was detected in lung samples collected on days 2 and 3 p.i. by both RRT-PCR and VI (Table 3).

**Table 3:** RRT-PCR and VI results in vaccinated turkeys infected with HPAI virus A/turkey/Italy/4580/1999 (H7N1)

Group	1 day p.i.	2 days p.i.	3 days p.i.	4 days p.i.	5 days p.i.
Virus isolation					
Breast	0/3	0/3	0/3	0/3	0/3
Thigh	0/3	0/3	0/3	0/3	0/3
Lung	0/3	1/3	2/3	0/3	0/3
Blood	0/15	0/12	0/9	0/6	0/3
RRT-PCR					
Breast	0/3	0/3	0/3	0/3	0/3
Thigh	0/3	0/3	0/3	0/3	0/3
Lung	0/3	1/3	2/3	0/3	0/3
Blood	0/15	0/12	0/9	0/6	0/3

Data presented as number positive/total.

#### Unvaccinated turkeys infected with LPAI virus

Birds showed mild respiratory disease with depression and sinusitis starting from day 3 p.i. (Table 4). The results of VI and RRT-PCR are presented in Table 5. Two blood samples collected on day 2 p.i. were positive by RRT-PCR but only one was confirmed by VI (viral titre <10^1^ EID_50_/0.1 ml). Despite this case of viraemia, no virus was isolated in muscles.

**Table 4:** Clinical signs and schedule of sacrificing and time of appearance of viraemia by RRT-PCR of unvaccinated turkeys infected with LPAI virus A/turkey/Italy/3675/1999 (H7N1)

Turkey number	1 day p.i.	2 days p.i.	3 days p.i.	4 days p.i.	5 days p.i.
341	Healthy	Killed humanely viraemic	–	–	–
342	Healthy	Healthy	Killed humanely	–	–
343	Healthy	Healthy	Sick/killed humanely	–	–
344	Healthy	Killed humanely viraemic	–	–	–
345	Healthy	Healthy	Sick	Sick/killed humanely	–
346	Healthy	Killed humanely	–	–	–
347	Healthy	Healthy	Sick	Killed humanely	–
348	Killed humanely	–	–	–	–
349	Healthy	Healthy	Sick	Sick	Killed humanely
350	Killed humanely	–	–	–	–
352	Healthy	Healthy	Healthy	Healthy	Killed humanely
353	Healthy	Healthy	Sick/killed humanely	–	–
354	Healthy	Healthy	Healthy	Sick	Killed humanely
355	Healthy	Healthy	Healthy	Killed humanely	–
400	Killed humanely	–	–	–	–

**Table 5:** RRT-PCR and VI results in unvaccinated turkeys infected with LPAI virus A/turkey/Italy/3675/1999 (H7N1)

Group	1 day p.i.	2 days p.i.	3 days p.i.	4 days p.i.	5 days p.i.
Virus isolation					
Breast	0/3	0/3	0/3	0/3	0/3
Thigh	0/3	0/3	0/3	0/3	0/3
Lung	0/3	2/3	3/3	0/3	0/3
Blood	0/15	1/12	0/9	0/6	0/3
RRT-PCR					
Breast	0/3	3/3	2/3	0/3	0/3
Thigh	0/3	2/3	2/3	0/3	0/3
Lung	0/3	3/3	3/3	0/3	0/3
Blood	0/15	2/12	0/9	0/6	0/3

Data presented as number positive/total.

#### Vaccinated turkeys infected with LPAI virus

No clinical signs were observed during the experimental trial and no virus was detected in lungs and muscles by either VI or RRT-PCR.

#### Avian influenza transmission-feeding test

No clinical signs were observed in any group fed on infective meat. Neither unvaccinated nor vaccinated birds showed increased antibody levels. All tracheal and cloacal swabs collected throughout the experiment yielded negative results.

## Discussion

The results of the challenge study with H7N1 HPAI indicate that this strain causes viraemia and is able to reach turkey muscle tissues. These data are in agreement with the evidence reported that in *Galliformes* species HPAI viruses cause viraemia and systemic infection and virus can be detected in the muscle tissues of infected birds. Several studies have shown that HPAI viruses may be recovered from meat of chickens, turkeys (Perkins & Swayne, 2001; Kishida *et al.*, 2004; Swayne & Beck, 2005; Swayne, 2006) and ducks (Tumpey *et al.*, 2002, 2003; Lu *et al.*, 2003; Beato *et al.*, 2007) following both field and experimental infection.

In addition to the existing data we evaluated the trend of viraemia, showing high levels of viable virus in blood during the first 3 days p.i. with HPAI virus, which correspond to high levels of virus in muscles.

With reference to the H7N1 LPAI virus reported here, infection results show the ability of this strain to cause viraemia in turkeys, although in a limited number of birds. This information is in contrast with the general consensus that LPAI viruses cause a strictly localized infection without viraemia, and is in keeping with the clinical condition developed by some birds following natural and experimental infection. There is only one precedent report of LPAI virus causing viraemia: Kishida *et al*. (2004]) described the isolation of two H9N2 strains from imported chicken meat and bone marrow. In their investigation, Kishida *et al*. were able to detect virus in blood, bone marrow and muscles of chickens infected experimentally with those viruses. Our findings differ from those obtained by Swayne & Beck (2005), who failed to detect LPAI viruses (H7N2) in the blood of chickens infected experimentally. However, this difference could be due to the strain variability and to host susceptibility.

In any case, despite the detection of viable virus from the blood, no virus was isolated from muscles of the unvaccinated LPAI-infected turkeys, although positive RRT-PCR results suggest that virus can reach muscle tissues.

In the present paper we report the absence of detectable virus in the blood and in meat of vaccinated turkeys infected with both LPAI and HPAI viruses. This evidence suggests that vaccination is an effective tool for preventing viraemia and, as a consequence, preventing meat from infected turkeys being infective.

Under experimental conditions, transmission of HPAI infection to naïve or vaccinated birds through the administration of 2 g meat homogenate containing 10^3.6^ EID_50_/0.1 g failed, and therefore no transmission of infection could be expected from meat of vaccinated birds that, both by RRT-PCR and by VI, appeared to contain no virus.

It is noteworthy that viable virus was recovered from the lungs of vaccinated turkeys challenged with HPAI virus. These data are in agreement with studies conducted by Swayne & Beck (2005), who detected viable LPAI H7N2 subtype virus from fluid collected post evisceration following internal body cavity wash. These findings suggest that contamination of the carcass with internal organ exudates could represent a means by which LPAI can be introduced into a country through legal trade. It can be concluded that even if no virus was detected in muscle tissue of vaccinated and challenged turkeys, bird carcasses can still represent a potential vehicle of infection if the hygienic conditions at the slaughterhouse are poor. For these reasons it appears reasonable to recommend that in poultry slaughter-houses special attention should be paid to treat carcass wash fluids properly and to completely eviscerate carcasses. It would, in any case, be logical to assume that the titre of the contaminating virus would not be high enough to generate a secondary cycle of infection if it were introduced in the animal food chain as swill feeding, giving the low initial titres and the dilution effect occurring during processing.

Several experimental studies highlight the possibility of AI transmission through the consumption of untreated infective meat. Purchase (1931) and Swayne & Beck (2005) showed that chickens fed on muscle tissues from HPAI-infected birds can become infected. On the basis of these previous investigations we tested the role that untreated infective meat may play in the transmission of AI viruses to vaccinated and unvaccinated chickens and turkeys. The results obtained with the transmission-feeding test indicate that infection by feeding birds with infective meat was achieved in neither unvaccinated nor vaccinated birds, and it can be suggested that a higher dose of virus is required to cause the infection by the oral route.

The results reported in the present study suggest that vaccination prevents viraemia and viral localization in turkey muscles and thus minimizes the potential of meat as vehicle for transmission of HPAI and LPAI viruses of the H7 subtype.
